# Exopolysaccharide producing rhizobacteria and their impact on growth and drought tolerance of wheat grown under rainfed conditions

**DOI:** 10.1371/journal.pone.0222302

**Published:** 2019-09-12

**Authors:** Naeem Khan, Asghari Bano

**Affiliations:** Department of Biosciences, University of Wah, Wah Cantt., Pakistan; ICAR-Indian Institute of Agricultural Biotechnology, INDIA

## Abstract

The demand for agricultural crops continues to escalate with an increasing population. To meet this demand, marginal land can be used as a sustainable source for increased plant productivity. However, moisture stress not only affects crop growth and productivity but also induces plants’ susceptibility to various diseases. The positive role of plant growth hormone, salicylic acid (SA), on the defence systems of plants has been well documented. With this in mind, a combination of plant growth promoting rhizobacteria (PGPR) and SA was used to evaluate its performance on wheat grown under rainfed conditions (average moisture 10–14%). The selected bacterial strains were characterized for proline production, indole-3-acetic acid (IAA), hydrogen cyanide (HCN), ammonia (NH_3_), and exopolysaccharides (EPS). Wheat seeds of two genotypes, Inqilab-91 (drought tolerant) and Shahkar-2013 (drought sensitive), which differed in terms of their sensitivity to drought stress, were soaked for three hours prior to sowing in 24-hour old cultures of the bacterial strains *Planomicrobium chinense* strain P1 (accession no. MF616408) and *Bacillus cereus* strain P2 (accession no. MF616406). SA was applied (150 mg/L), as a foliar spray on one-month-old wheat seedlings. A significant reduction in the physiological parameters was noted in the plants grown in rainfed conditions but the PGPR and SA treatment effectively ameliorated the adverse effects of moisture stress. The wheat plants treated with PGPR and SA showed significant increases in leaf protein and sugar contents and maintained higher chlorophyll content, chlorophyll fluorescence (fv/fm) and performance index (PI) under rainfed conditions. Leaf proline content, lipid peroxidation, and antioxidant enzyme activity were higher in the non-inoculated plants grown in rainfed conditions but significantly reduced in the inoculated plants of both genotypes. Integrative use of a combination of PGPR strains and SA appears to be a promising and eco-friendly strategy for reducing moisture stress in plants.

## Introduction

The current scenario of climate change has resulted in global warming coupled with infrequent precipitation, subsequently increasing the demand for irrigation [1]. Rainfed soils are incapable of moving water from deeper layers of soils through capillary action. These soils have a poor structure and exhibit lower water holding capacity, with fewer nutrients; however, the fertility status and yield capabilities of these soils can be improved by applying compost leaf mold, manure or biofertilizer [2–3]. There is an increasing demand to improve tolerance against drought in wheat, in order to fulfil food demand worldwide [4–5].

Bacteria that live in the soil and interact with plants and enhance their growth directly or indirectly are known as plant growth promoting rhizobacteria (PGPR). PGPR improve the root growth, thereby enhancing the accessibility of micro-nutrients to the plant roots [6]. Plant roots produce an array of organic compounds that are an efficient source of carbon inside the soil [7–8]. These compounds are secreted from the roots as exudates; they attract soil microbes, including PGPR [9, 10]. Rhizobacteria maintain the water budget of plants under rainfed conditions by improving the growth of the root system [11]. Furthermore, PGPR improves the water use efficiency (WUE) and augments the water absorption ability of roots under conditions of water scarcity [12]. Inoculation of plants with PGPR increases the growth rate and fosters seedling emergence; it also imparts tolerance to various stresses and plant pathogens. A PGPR-induced increase in the development and yield of crops has been demonstrated in both greenhouse and field trials [13–16]. Several mechanisms have been proposed for the action of PGPR [17]. Some mechanisms produce different types of plant metabolites, such as HCN, 2,4-diacetylphloroglucinol (DAPG) [18], antibiotics, e.g. phenazine [19], and volatile compounds that motivate plant growth [20]. Other strains produce exopolysaccharides, siderophores, biofilm and plant hormones which influence plant physiological processes [21].

Salicylic acid is known for its defensive role when present in plants under appropriate concentrations; it also plays a key role in the plant development process by modulating plant responses to abiotic stresses [22–27]. Foliar spray of SA ameliorates the negative effects of drought and increases the restoration process in plants [28–31]. The present study aims to evaluate the role of *Planomicrobium chinense* and *Bacillus cereus* alone and in combination with SA, on various metabolites, physiology and drought tolerance of wheat grown under rainfed conditions.

## Materials and methods

### Experimental work plan

The experiment was conducted using wheat plants grown under a natural rainfed conditions in a field. Seeds were grown in the rainfed area of Kohi Barmol (14% soil moisture content), located 35 km from Mardan. Mardan is located in Pakistan, in the south western part of Khyber Pakhtunkhwa Province (at 34°12'0N 72°1'60E and at an altitude of 283 m). Mardan has a hot semi-arid climate with an average temperature of 22.2 °C. October is the driest month, with an average rainfall of 12 mm. August is the wettest month, with an average rainfall of 122 mm. Seeds of two wheat genotypes i.e. Inqilab-91 (drought tolerant) and Shahkar-2013 (drought sensitive) were gained from Ayub Agriculture Research Institute, Faisalabad. The experiment was conducted using a Randomized Complete Block Design (RCBD) with a plot size of 5 ×1. 5 m, and a row-to-row distance of 40 cm. Four replicates were used per treatment.

**The experiment consisted of five treatments as described below**:

T1_-_ Irrigated control plants (+ve control)
T1_-_ Seeds grown under rainfed conditions (-ve control)
T3_-_ Seeds grown under rainfed conditions and inoculated with broth culture of Planomicrobium *chinense* and *Bacillus cereus*.
T4_-_ Seeds grown under rainfed conditions and treated with the consortium of 2-PGPR and sprayed with SA at 3 leaf-stage.
T5_-_ Seeds grown under rainfed conditions and then plants were sprayed with SA at 3 leaf-stage.

### Sterilization of the seeds

Prior to seed inoculation at room temperature, the seeds were surface sterilized with ethanol (70%) for 3 min, and then soaked in Clorox (10%) for 2–3 min after which they were successively washed with pure water.

#### Method of inoculation

The LB broth was inoculated with fresh (24 h old) bacterial culture and incubated in a shaker for 48 h at 27 °C followed by centrifuging at 10,000rpm for 10 min. Pellets were mixed with distilled water after removal of supernatant and the optical density (at 660 nm) was adjusted to 1. The seeds were then soaked in the broth for 3 h before sowing.

### Characterization of bacterial isolates for beneficial traits to promote plant growth

#### IAA production by selected PGPR strains

The amount of IAA produced by each strain was determined by the method of Salkowski’s reagent [32].

#### HCN production by selected PGPR strains

The amount of HCN produced by each strain was calculated by using the method reported in Lorck [33]. For this purpose the selected strains were grown in King’s B broth supplemented with glycine (4.4 g/ l) and the absorbance was read at 625 nm [34].

#### NH_3_ production by selected PGPR strains

Cappuccino and Sherman [35] procedure was followed for determination of NH_3_. The bacterial isolates were grown in the peptone water broth for 48–72 h at 30°C. After centrifugation, the bacterial supernatant was mixed with Nessler’s reagent. Development of brown to yellow colour indicates the presence of ammonia.

#### Exopolysaccharides production by PGPR

The bacterial strains were cultured in optimized mineral salts medium with 12.6% K_2_HPO_4_, 18.2% KH_2_PO_4_, 10% NH_4_NO_3_, 1% MgSO_4_.7H_2_O, 0.6% MnSO_4_, 1% CaCl_2_.2H_2_O, 0.06% FeSO_4_.2H_2_O, 1% sodium molybdate, 1.5% NaCl, and 0.2% of glucose in 1L of distilled water for 10 days [36]. After incubation for 10 d, the bacterial cultures (250 ml) were centrifuged at 15,000 rpm for 20 min at 4°C. The EPS were extracted from the supernatant by the addition of twofold ice cold ethanol (95%), the solution was chilled at 4°C for complete precipitation. EPS were collected from above solution [37]. Extracted EPS were lyophilized with Labonco lyophilizer at 3000 psi and stored at room temperature [36]. To determine the solubility of EPS, small quantities of lyophilized EPS were suspended in 2 ml of benzene, water, chloroform, acetone, ethanol, and methanol. The mixture was vortexed and allowed to stabilize for some time and the pellet formation was observed.

### Biochemical analyses of wheat

#### Leaf chlorophyll content, chlorophyll fluorescence and performance index

Chlorophyll content was determined with the help of a portable soil plant analysis development (SPAD) chlorophyll meter whereas chlorophyll fluorescence and performance index was measured using fluorometer.

#### Leaf protein content

Standard procedure of Lowry *et al*. [38] was followed for the determination of soluble protein content in the leaves of crop plants using BSA (bovine serum albumen) as standard.

#### Sugar estimation

For estimation of sugar in the leaves of wheat plants the method of Dubois *et al*. [39] was followed. Leaf tissues from fresh plant was grounded in distilled water followed, by centrifugation at 4000 rpm. The supernatant obtained as a result of centrifugation was mixed with concentrated sulphuric acid (5 ml) and phenol (1 ml; 80%) and their absorbance was noted at 420 nm. The concentration of sugar in unknown sample was calculated with reference to standard curve made from glucose:

Sugarcontent(mg/g)=Kvalue×Dilutionfactor×Absorbance/Samplewt.

K value = 20, Dilution factor = 10, Weight of sample = 0.5 g.

#### Leaf proline content

Leaf proline content was determined following the method of Bates *et al*. [40].

#### Lipid peroxidation and total phenolic content

For determination of lipid peroxidation the amount of malondialdehyde (MDA) produced by the reaction of thiobarbituric acid (TBA) as defined by Li [41] was followed whereas, Folin-Ciocalteu colorimetric method [42] was used for the estimation of total phenolic content.

#### Antioxidant enzymes activity

For determination of antioxidant enzymes activity, fresh leaves (0.5 g) were crushed in phosphate buffer (5 ml) while keeping on ice bath. The obtained homogenate was centrifuged for 22 minutes at 13000 g at 4 °C. The supernatant obtained was used to study the antioxidant enzymes activity including peroxidase [43–44], ascorbate peroxidase [45], catalase [46] and superoxide dismutase [47].

#### Shoot and root fresh and dry weights

The shoot fresh weight of five plants and their roots were measured with the help of an electronic balance. The selected shoots and roots were then oven dried at 70°C for determination of dry weights.

### Relative Water Content (RWC)

Relative Water Content (RWC) of leaves was determined following the formula suggested by Weatherly [48]. The fresh weight of leaves was recorded for each treatment separately. The leaves were than separately kept by dipping in distilled water in test tubes and then left for about 24 h. Within this time the leaves were fully turgid and were weighed again. After weighing the same leaves were placed in oven at 70 °C for 72 h. After 72 h the dry weight of leaves was measured.

RWC=[(freshmass−drymass)/(saturatedmass−drymass)]×100.

### Soil moisture content (%)

Soil moisture content was calculated as:

100×[(W1−W2)/W2]

where W1 = fresh soil weight, W2 = oven dried weight of 3 kg of field soil [49].

### Nutrients analysis of rhizosphere soil

The rhizophere soil was examined for various micro- and macronutrients following the method developed by Soltanpour and Schwab [50].

### Data analysis

The obtained data was analysed with Statistics (version. 8.1). ANOVA was done to determine the effect of different treatments and associated errors. To classify significant variances among treatments, a mean comparison of trait was carried out by using protected LSD (*P = 0*.*05*) test where error mean square was used to assess the standard error alterations between mean.

## Results

### Biochemical characteristics of PGPR strains

The two PGPR strains were tested for the production of proline, IAA, HCN, and NH_3_. Maximum proline and IAA production (1.432 μg/mg and 0.491 μg/ml) were recorded in *Bacillus cereus*, followed by *Planomicrobium chinenese* (1.246 μg/mg and 0.469 μg/ml) whereas, *Planomicrobium chinense* was more effective for HCN production. The two selected PGPR isolates were checked for the production of NH_3_ and HCN and found that both of them were positive for NH_3_ and HCN production. In quantitative analysis, *Planomicrobium chinense* was found most effective with maximum O.D value of 0.071 followed by *Bacillus cereus* (0.051) for the production of HCN (Table 1).

**Table 1:** Proline, IAA, HCN production by selected PGPR strains and detection of NH_3_.

Selected PGPR Strains	Proline Production (μg/mg)	IAA Production (μg/ml)	HCN production	NH_3_ Detection
*Planomicrobium chinense*	1.246	0.469	0.071	+
*Bacillus cereus*	1.432	0.491	0.051	+

+ Present.

#### Solubility and chemical composition of exopolysaccharides (EPS) extracted from PGPR

The lyophilized EPS was soluble in water and insoluble in benzene, acetone and chloroform (Table 2). The chemical composition of PGPR-induced EPS shows that the EPS produced by *P*. *chinense* had a higher percentage of sugar and protein (i.e. 97% and 98% respectively) as compared to control. The highest uronic acid concentration (95%) was noted in the EPS of *B*. *cereus* as compared to control sample.

**Table 2:** Chemical characterization of exopolysaccharides (EPS).

PGPR Strains	Sugar content (μg/g)	Protein content (μg/g)	Uronic acid (μg/mg)
Control	215.5	16.1	0.076
*Planomicrobium chinense*	6672.3	835.3	1.33
*Bacillus cereus*	5435.5	699.9	1.49

### Biochemical characters

#### Chlorophyll content and performance index (PI)

**Fig 1:**
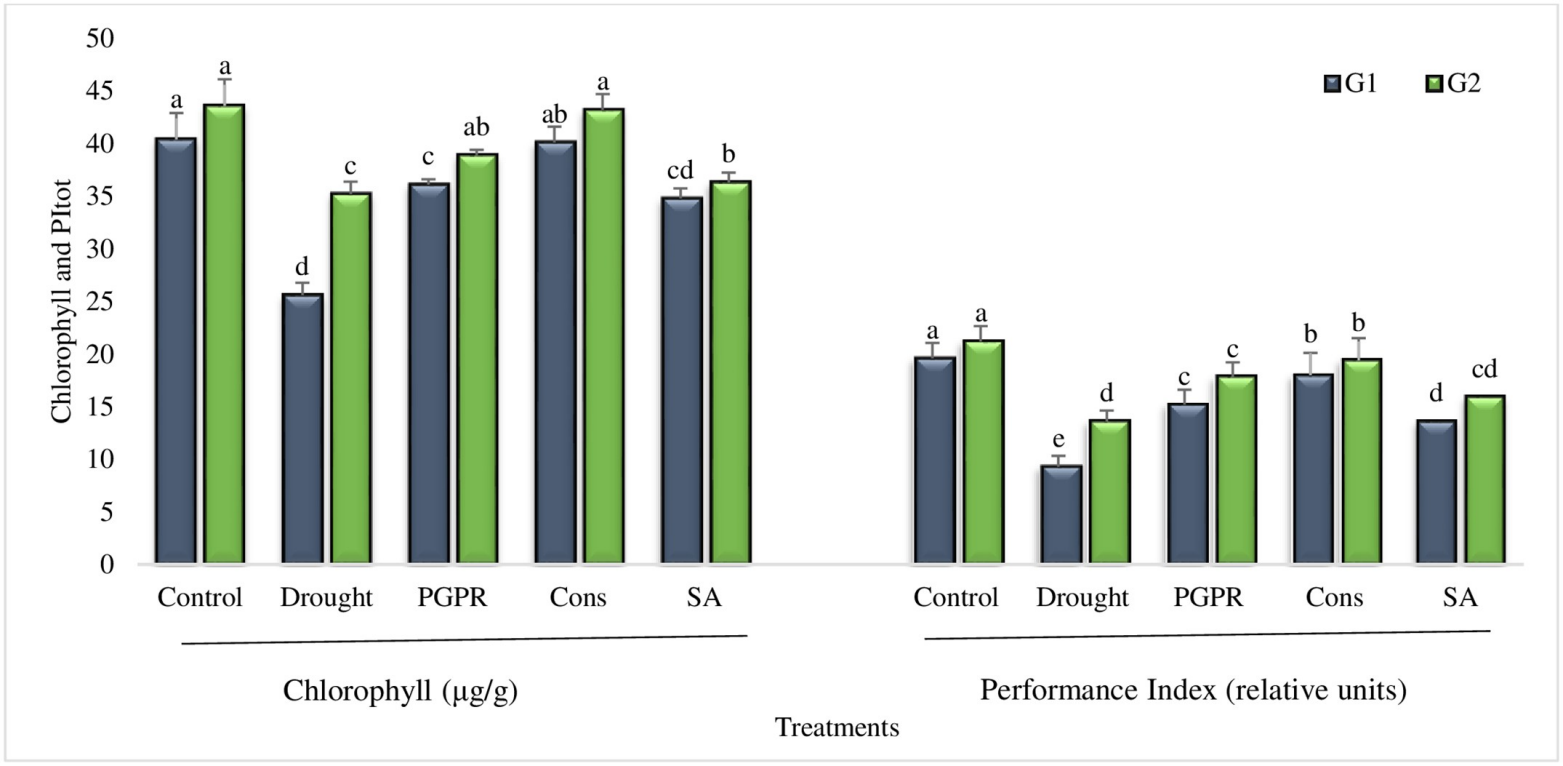
Chlorophyll content and performance index of drought sensitive (G1) and tolerant (G2) wheat genotypes grown under rainfed field condition. Data are means of four replicates along with standard error bars. Different significance levels were denoted with different letters. For treatments with the same letter, the difference is not statistically significant and those with a different letter, the difference is statistically significant (*P < 0*.*05*) within treatments.

The chlorophyll content was decreased in plants grown in rainfed conditions. The percentage of decrease in the chlorophyll content of the untreated tolerant genotype was less (19%) as compared to that of the sensitive genotype (36%). In comparison to untreated control plants grown under rainfed conditions, the chlorophyll content was improved by all the treatments in both genotypes (Fig 1). Maximum increases (36% and 19%) were observed in plants receiving combined treatment of PGPR and SA (T4). The coinoculation of 2-PGPR (T3) significantly reduced (upto 25%) the drought induced-damage to chlorophyll content in the sensitive genotype. Foliar application of SA exerted a positive effect on wheat seedlings and minimized the drought stress induced % damage in chlorophyll content (upto 21%). The performance index (PI), which is a sensitive indicator of water stress in wheat was also reduced significantly (53%) in sensitive genotype grown under rainfed conditions. The tolerant genotype was able to maintain a higher PI (54% greater than the sensitive genotype) under rainfed conditions. The cmbination of PGPR and SA significantly enhanced (48% and 30%) the PI of both the sensitive and tolerant genotypes respectively. The SA alone significantly enhanced (32% and 14%) the PI of both the genotypes as compared to untreated plants grown under rainfed conditions (T2) (Fig 1).

#### Chlorophyll fluorescence (Fv/Fm)

**Fig 2:**
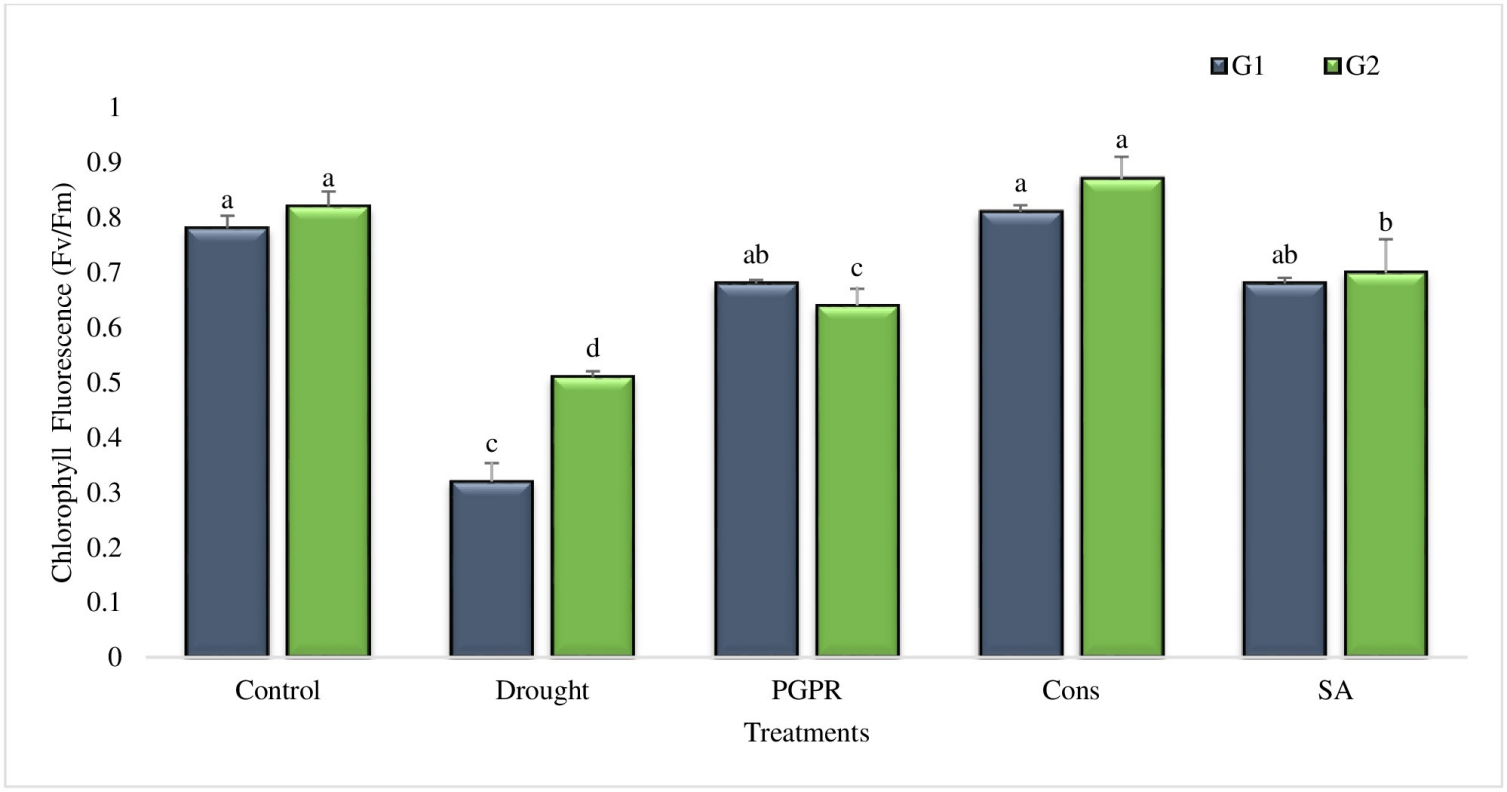
Chlorophyll fluorescence in the leaves of drought sensitive (G1) and tolerant (G2) wheat genotypes grown under rainfed field condition. Data are means of four replicates along with standard error bars. Different significance levels were denoted with different letters. For treatments with the same letter, the difference is not statistically significant and those with a different letter, the difference is statistically significant (*P < 0*.*05*) within treatments.

The photochemical efficiency of PSII (Fv/Fm) showed variability between genotypes and treatments (Fig 2). The Fv/Fm ratio was reduced for both genotypes under rainfed conditions but the reduction was greater (59%) in the sensitive genotype. The combination of PGPR and SA significantly enhanced the Fv/Fm ratio and the percent increase was even greater than that of irrigated control. The coinoculation of *P*. *chinense* and *B*. *cereus* significantly increased Fv/Fm ratio, the response was greater in the sensitive genotype. Salicylic acid alone significantly enhanced (36% and 27%) the Fv/Fm ratio of both genotypes as compared to untreated plants grown under rainfed conditions.

#### Leaf protein and sugar contents

**Fig 3:**
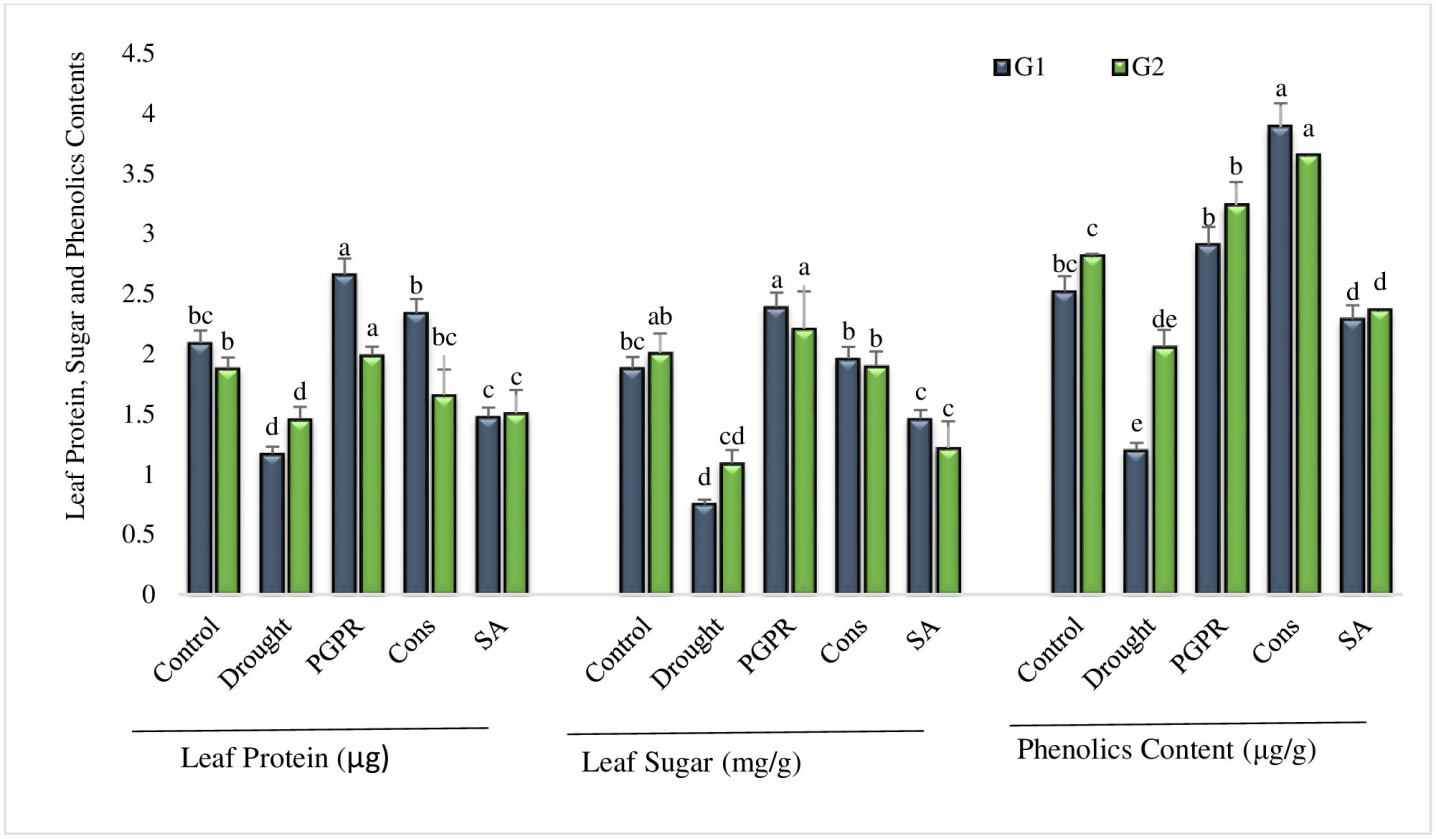
Leaf protein, sugar and phenolic contents of drought sensitive (G1) and tolerant (G2) wheat genotypes grown under rainfed field condition. Data are means of four replicates along with standard error bars. Different significance levels were denoted with different letters. For treatments with the same letter, the difference is not statistically significant and those with a different letter, the difference is statistically significant (*P < 0*.*05*) within treatments.

The result showed that the combined treatment of *P*. *chinense* and *B*. *cereus* (T3) had significantly (56% and 27%) enhanced the leaf protein content over uninoculated untreated plants grown under rainfed conditions, followed by their combined treatment with SA (T5) (Fig 3). Foliar application of SA alone or in combination with PGPR was not significantly effective for accumulation of leaf protein content. As compared to control plants grown under drought, leaf sugar content was increased in all the treatments of both the genotypes. The combined treatment of *P*. *chinense* and *B*. *cereus* (T3) was most effective in sensitive genotype and significantly (69%) enhanced the leaf sugar content under rainfed conditions. SA applied singly (T5) was also more effective in sensitive genotype and significantly enhanced (36%) the leaf sugar content (Fig 3).

#### Phenolic content of leaves

The phenolic contents of leaves of both the genotypes were significantly reduced under rainfed conditions but the effect was more pronounced in sensitive genotype however, the application of PGPR and SA (T4) significantly reduced the percent decrease in leaf phenolic content. Maximum increase (69% and 44%) in phenolic content was recorded in the leaves of plants treated with PGPR and SA (T4). The coinoculation of both the PGPR (i.e. *P*. *chinese* and *B*. *cereus*) increased the phenolic content even greater than the irrigated control. The combined treatment (T4) of PGPR and SA was more responsive in sensitive genotype whereas, the coinoculation of 2-PGPR (T3) was more effective in tolerant genotype. Salicylic acid alone had also significantly enhanced (48%) the phenolic content of sensitive genotype but was less effective (13%) in tolerant genotype (Fig 3).

#### Leaf proline content

**Fig 4:**
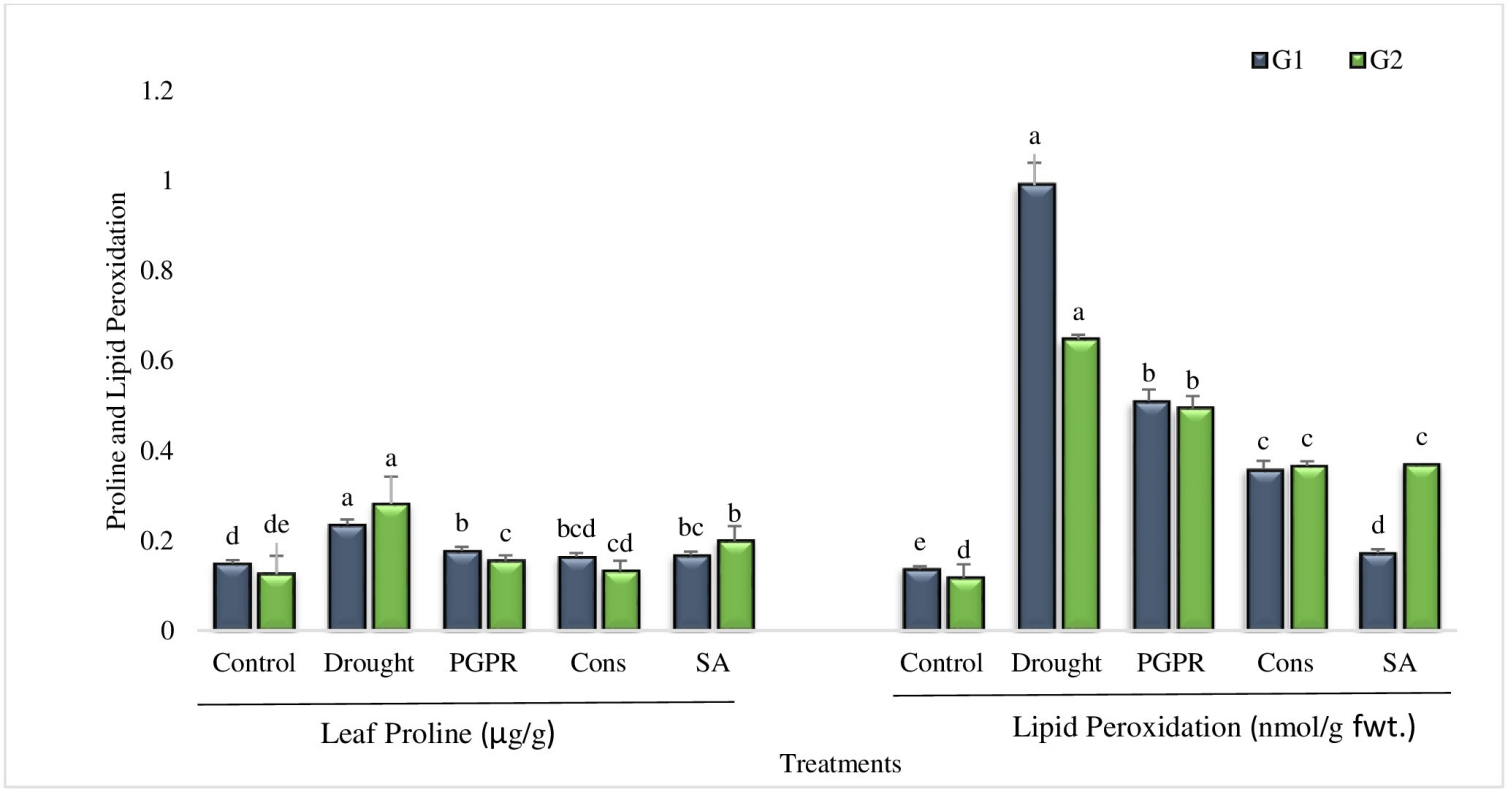
Leaf proline content and lipid peroxidation in the leaves of drought sensitive (G1) and tolerant (G2) wheat genotypes grown under rainfed field condition. Data are means of four replicates along with standard error bars. Different significance levels were denoted with different letters. For treatments with the same letter, the difference is not statistically significant and those with a different letter, the difference is statistically significant (*P < 0*.*05*) within treatments.

The result revealed a significant decrease in leaf proline content as compared to stress control (T2), though the values were greater than irrigated control (T1) (Fig 4). The combination of PGPR and SA (T4) was the most effective treatment that significantly reduced (30% and 53%) the leaf proline content in both the genotypes grown under rainfed conditions. The coinocualtion of *P*. *chinense* and *B*. *cereus* were also significantly reduced the leaf proline content. The untreated tolerant genotype displayed enhanced proline accumulation over sensitive genotype under rainfed conditions. The foliar application of SA (T5) resulted 29% decrease in the leaf proline content of both the genotypes.

#### Lipid peroxidation

Substantial reduction in lipid peroxidation was recorded in all the treatments in comparison to untreated plants grown under rainfed conditions (T2) (Fig 4). Highly significant decrease (82%) in lipid peroxidation of sensitive genotype was noted in plants treated with SA (T5) whereas, in tolerant genotype the maximum decrease (43%) was noted in the combined treatment (T4) of PGPR plus SA, followed by the foliar application of SA (T5). In general, the lipid peroxidation was greater in tolerant genotype when treated with SA under rainfed conditions.

#### Antioxidant enzymes activity

**Fig 5:**
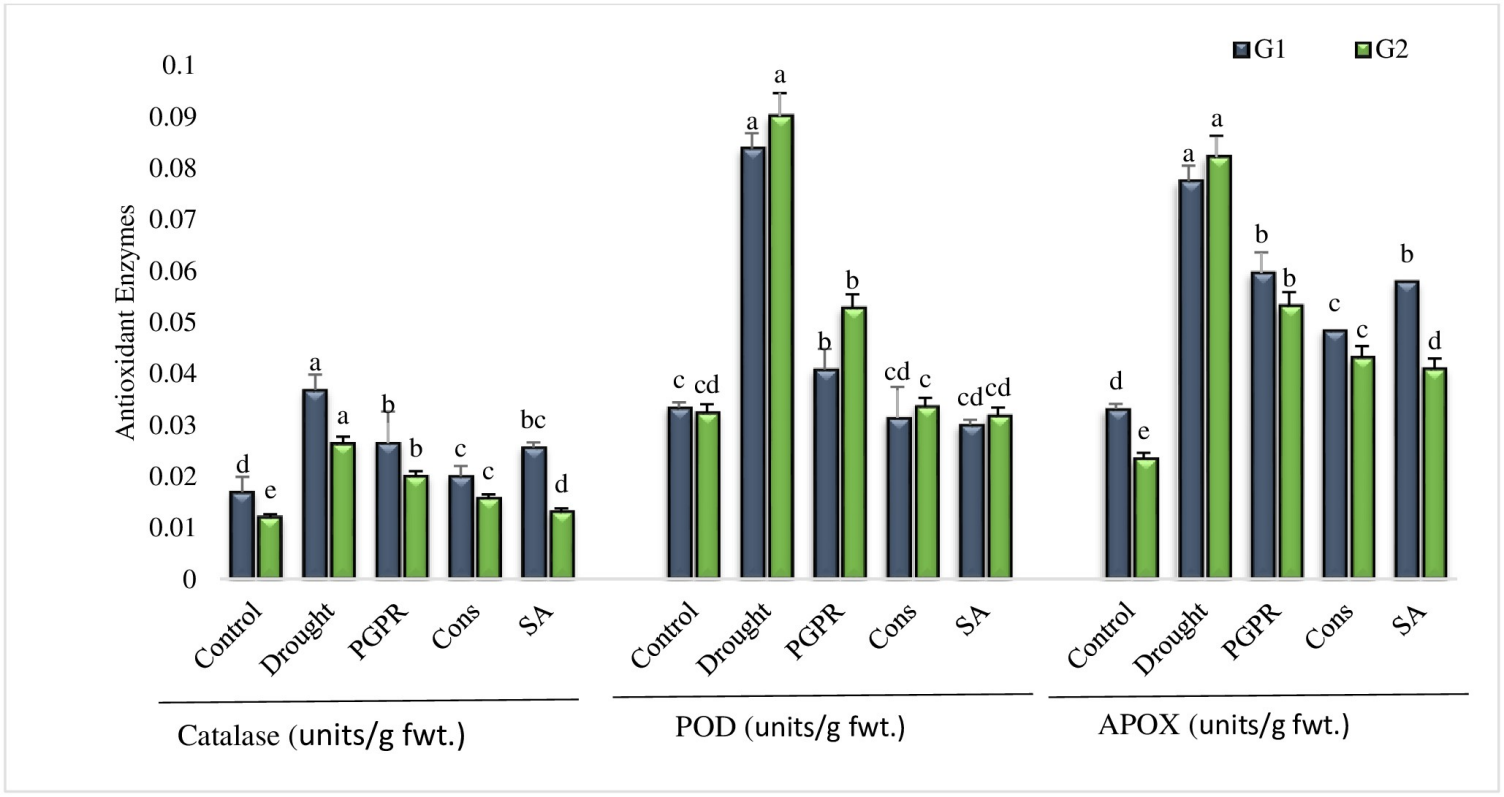
Antioxidant enzymes activities in the leaves of drought sensitive (G1) and tolerant (G2) wheat genotypes grown under rainfed field condition. Data are means of four replicates along with standard error bars. Different significance levels were denoted with different letters. For treatments with the same letter, the difference is not statistically significant and those with a different letter, the difference is statistically significant (*P < 0*.*05*) within treatments.

Plants growing in rainfed conditions showed significant increase in the activities of all the antioxidant enzymes (i.e. CAT, POD and APOX). The inoculated plants showed decrease in the antioxidant enzymes activity over control. The foliar application of SA (T5) was most effective treatment in reducing the activities of antioxidants in tolerant genotype and significantly reduced the catalase (50%), POD (65%) and APOX (50%) activities as compared to untreated plants grown under rainfed conditions. The combined treatment of PGPR and SA (T4) had significantly decreased (46%, 63% and 38%) the activities of antioxidant enzymes in the sensitive genotype. The activity of catalase was higher in all the treatments of the sensitive genotype whereas, higher POD values were noted in the tolerant genotype (Fig 5).

#### Shoot and root fresh weights

**Fig 6:**
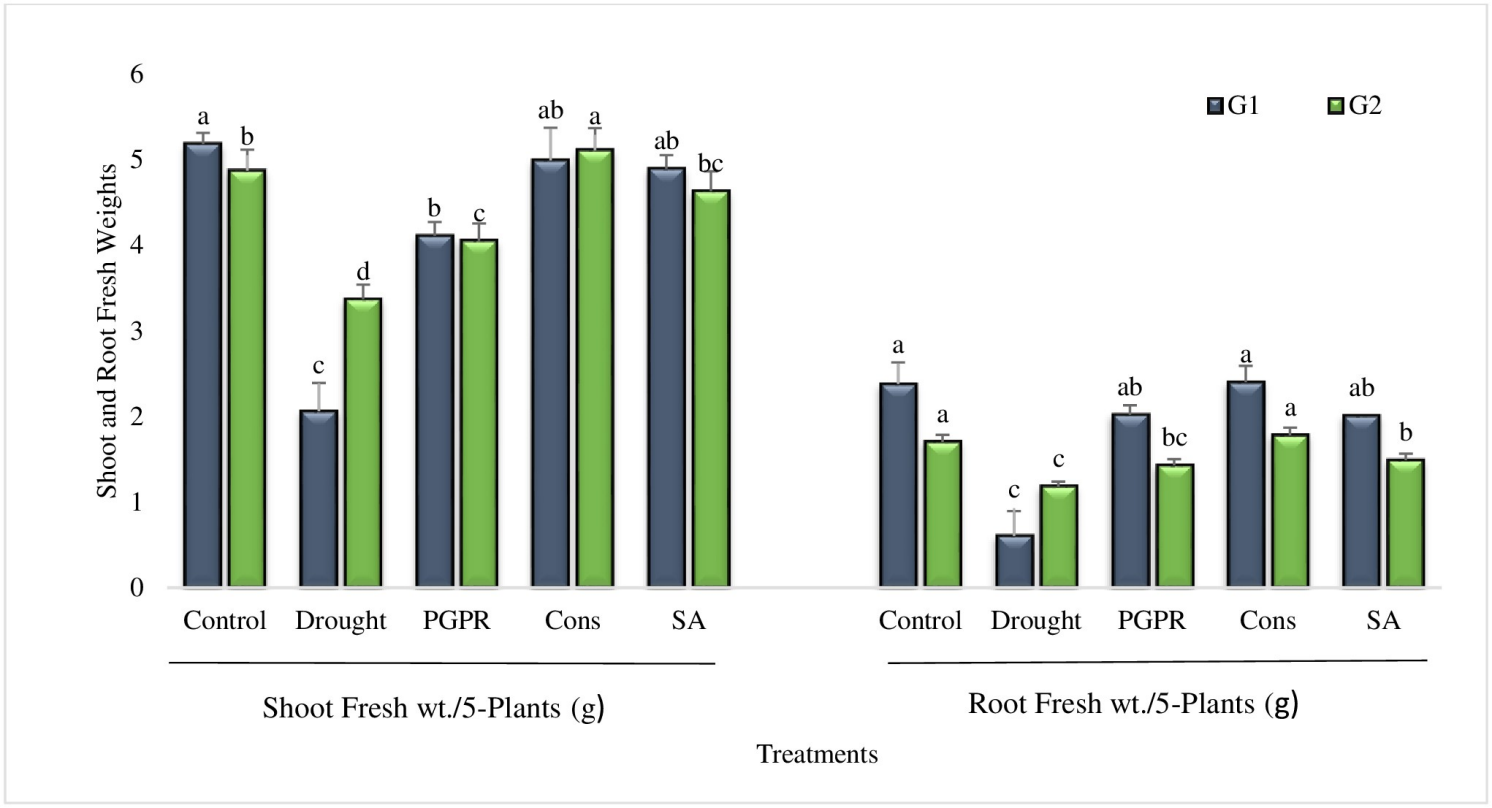
Shoot and root fresh weights of drought sensitive (G1) and tolerant (G2) wheat genotypes grown under rainfed field condition. Data are means of four replicates along with standard error bars. Different significance levels were denoted with different letters. For treatments with the same letter, the difference is not statistically significant and those with a different letter, the difference is statistically significant (*P < 0*.*05*) within treatments.

It in inferred from results that shoot fresh weight was considerably improved in all the treatments over untreated plants gown under rainfed conditions (Fig 6). Greater increase (59% and 34%) in shoot fresh weight was noted as a result of combined application of PGPR with SA (T4). The combined treatment of PGPR and SA (T4) resulted higher increase in the shoot fresh weight of tolerant genotype; the increase was much greater than that of irrigated control. Salicylic acid significantly enhanced (58% and 27%) the shoot fresh weight and the increase was even greater than that of coinoculation of *P*. *chinenes* and *B*. *cereus* (T3). PGPR inoculation also augmented the root fresh weight in comparison to untreated plants grown in rainfed conditions though, the increase was less than irrigated control in most of the treatments. Maximum increase (75% and 34%) in root fresh weight was recorded in combined treatment of PGPR and SA (T4) in both the sensitive and tolerant genotypes, respectively. *Planomicrobium chinense* in combination with *B*. *cereus* (T3) was more effective in sensitive genotype.

#### Shoot and root dry weights

**Fig 7:**
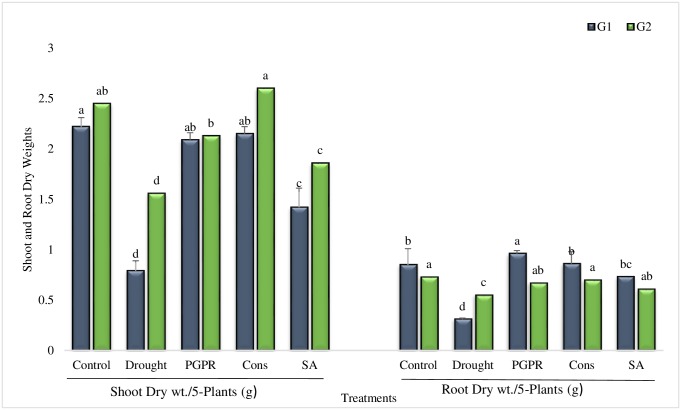
Shoot and root dry weights of drought sensitive (G1) and tolerant (G2) wheat genotypes grown under rainfed field condition. Data are means of four replicates along with standard error bars. Different significance levels were denoted with different letters. For treatments with the same letter, the difference is not statistically significant and those with a different letter, the difference is statistically significant (*P < 0*.*05*) within treatments.

Shoot dry weight was highly significantly increased in all the inoculated treatments over untreated plants grown under rainfed conditions (Fig 7). Maximum increase (63% and 40%) in shoot dry weight was recorded in the combined treatment of PGPR and SA (T4). The consortium treatment was more effective in tolerant genotype than sensitive genotype and resulted greater increase, even greater than irrigated control. The coinoculation of *P*. *chinense* and *B*. *cereus* was par with both the genotypes. Salicylic acid alone (T5) had also significantly enhanced (44%) the shoot dry weight of sensitive genotype whereas it was less effective in tolerant genotype (14%). Similarly, root dry weight was increased in all the treatments over untreated plants grown under rainfed conditions. Maximum increase (67%) in root dry weight of sensitive genotype was recorded in combined treatment of *P*. *chinense* and *B*. *cereus* (T3) whereas, the higher increase (21%) in root dry weight of tolerant genotype was noted in plants treated with the consortium of PGPR and SA (T4). Rainfed conditions resulted 64% reduction in root dry weight of sensitive genotype however, the application of SA significantly overcame drought induced decrease in the root dry weight.

### Relative Water Content

The relative water content of both the genotypes was significantly reduced under moisture stress. However, the percent damage was more in sensitive genotype (72%) as compared to that of tolerant genotype (31%). The application of PGPR alone (T3) or in combination with SA (T4) significantly reduced the percent damage due to moisture stress and significantly enhanced the RWC of both the genotypes. Highly significant increase (70% and 28%) in Relative Water Content was recorded in plants treated with PGPR and SA (T4). The Relative Water Content of tolerant genotype was 61% higher than sensitive genotype. Salicylic acid, had significantly enhanced (67% and 17%) the Relative Water Content of both the genotypes respectively and the percent increase was even greater than plants coinoculated with *P*. *chinense* and *B*. *cereus* in sensitive genotype (Table 3).

**Table 3:** Relative Water Content of plant shoot and soil moisture content of rhizosphere soil.

Treatments	RWC (S)	RWC (T)	SMC (S)	SMC (T)
**T1**	77.1^a^ (±2.08)	80.8^a^ (±1.73)	1.25^c^ (±0.013)	0.93^c^ (±0.021)
**T2**	21.9^e^ (±0.88)	56.1^e^ (±0.64)	0.44^e^ (±0.018)	0.34^d^ (±0.023)
**T3**	66.4^cd^ (±1404)	73.1^c^ (±0.32)	1.34^b^ (±0.043)	1.46^bc^ (±0.026)
**T4**	71.9^b^ (±2.01)	77.5^b^ (±1.27)	1.37^a^ (±0.061)	1.49^a^ (±0.052)
**T5**	67.2^c^ (±1.16)	67.3^d^ (±0.49)	1^d^ (±0.036)	1.15^b^ (±0.038)

T1-Irrigated control, T2-Untreated plants grown under rainfed conditions, T3-Coinouclation of *P*. *chinense* and *B*. *cereus*, T4-Consortium of PGPR and SA, T5-SA only. Different significance levels were denoted with different letters (a-e). Different letter shows that the difference is statistically significant (*P* < 0.05) within treatments.

#### Soil moisture content

The moisture content of the rhizosphere soil was improved following PGPR inoculation. Increases were higher (68% and 77% over control) in the rhizosphere of plants treated with PGPR and SA (T4) in both the genotypes respectively. The combined treatment of *P*. *chinense* and *B*. *cereus* was also effective and significantly improved the moisture content of the rainfed soil. (Table 3).

#### Macronutrient accumulation in the rhizosphere soil

All the treatments increased Ca content in wheat rhizosphere compared to the rhizosphere of uninoculated plants grown under rainfed conditions. Combination of PGPR and SA (T4) resulted maximum increase in Ca accumulation in both the sensitive (42%) and tolerant (37%) genotypes over untreated plants grown under rainfed conditions. Coinoculation of *P*. *chinense* and *B*. *cereus* was also stimulatory but the percent increase was less. The combined treatment of *P*. *chinense* and *B*. *cereus* (T3) significantly increased (57%) the Mg content in the rhizosphere of sensitive genotype. The consortium of PGPR and SA (T4) further augmented (71%) Mg content in the rhizosphere of tolerant genotype. Salicylic acid enhanced (27% and 41%) the Mg accumulation in both the genotypes. The Na (69% and 59%) and K (50% and 51%) accumulation were markedly improved in T4 (combination of PGPR and SA). The macronutrient accumulation was higher for tolerant genotype than that of sensitive genotype grown under rainfed conditions (Table 4).

**Table 4:** Ca, Mg, Na, and K contents (mg/kg) in rhizosphere of wheat grown in rainfed conditions.

Treatments	Ca (S)	Ca (T)	Mg (S)	Mg T)	Na (S)	Na (T)	K (S)	K (T)
T1	1.43^d^ (±0.05)	1.75^e^ (±0.06)	1.09^d^ (±0.05)	1.12^d^ (±0.04)	1.57^c^±0.07	1.23^d^ (±0.06)	14.4^d^ (±0.41)	16.2^cd^ (±0.35)
T2	1.28^e^ (±0.04)	1.61^d^ (± 0.08)	0.96^de^ (±0.08)	0.92^e^ (±0.03)	0.89^e^ (±0.05)	1.05^e^ (±0.08)	12.2^de^ (±0.23)	13.4^e^ (±0.41)
T3	2.07^ab^ (±0.09)	2.38^ab^ (±0.09)	2.22^b^ (±0.04)	2.49^b (^±0.08)	1.81^b^ (±0.09)	1.89^b^ (±0.05)	21.7^b^ (±0.12)	23.7^b^ (±0.59)
T4	2.21^a^ (±0.07)	2.54^a^ (±0.05)	2.19^b^ (±0.05)	3.13^a^ (±0.02)	2.88^a^ (±0.19)	2.49^a^ (±0.1)	24.2^a^ ±0.48	27.5^a^ (±0.38)
T5	1.51^c^ (±0.09)	2.01^c^ (±0.05)	1.32^c^ (±0.08)	1.56^c^ (±0.06)	1.42^cd^ (±0.15)	1.33^c^ (±0.11)	17.7^c^ (±0.20)	18.8^c^ (±0.66)

T1-Irrigated control, T2-Untreated plants grown under rainfed conditions, T3-Coinouclation of *P*. *chinense* and *B*. *cereus*, T4-Consortium of PGPR and SA, T5-SA only. Different significance levels were denoted with different letters (a, b, c). Different letter shows that the difference is statistically significant (*P* < 0.05) within treatments and ± represents standard error values.

#### Micronutrient accumulation in the rhizosphere soil

The accumulation of micronutrients were significantly reduced in the rhizosphere soil of plants grown under rainfed conditions. Nevertheless, inoculation with rhizoabacteria significantly accumulated micronutrients even under severe rainfed conditions. Maximum increase (79% and 60%) in Cu accumulation and Cr accumulation (63% and 69%) were noted in the rhizosphere of plants treated with consortium of PGPR and SA (T4). Higher accumulation of Zn (82% and 84%) was recorded in the rhizosphere of wheat treated with consortium of PGPR and SA (T4) whereas, the coinoculation of *P*. *chinense* and *B*. *cereus* (T3) was most effective (47% and 50%) for Fe accumulation followed by the combined treatment (46%) of PGPR and SA (T4). (Table 5).

**Table 5:** Cu, Cr, Zn, and Fe contents (mg/kg) in rhizosphere of wheat grown in rainfed conditions.

Treatments	Cu (S)	Cu (T)	Cr (S)	Cr (T)	Zn (S)	Zn (T)	Fe (S)	Fe (T)
T1	0.025^c^ (±0.002)	0.034^d^ (±0.004)	0.042^d^ (±0.001)	0.038^c^ (±0.002)	0.039^d^ (±0.001)	0.039^d^ (±0.003)	5.7^c^ (±0.045)	5.51^c^ (±0.16)
T2	0.016^d^ (±0.001)	0.025^e^ (±0.002)	0.031^e^ (±0.002)	0.024^d^ (±0.0009)	0.022^e^ (±0.003)	0.022^e^ (±0.001)	3.22^e^ (±0.12)	3.21^e^ (±0.0087)
T3	0.054^b^ (±0.004)	0.046^bc^ (±0.003)	0.075^b^ (±0.004)	0.042^bc^ (±0.003)	0.098^b^ (±0.008)	0.09^b^ (±0.008)	6.12^a^ (±0.19)	6.45^a^ (±0.42)
T4	0.078^a^ (±0.006)	0.062^a^ (±0.003)	0.084^a^ (±0.006)	0.077^a^ (±0.005)	0.123^a^ (±0.008)	0.138^a^ (±0.007)	5.97^ab^ (±0.21)	5.97^ab^ (±0.33)
T5	0.053^b^ (±0.002)	0.051^b^ (±0.003)	0.059^c^ (±0.004)	0.046^b^ (±0.004)	0.082^c^ (±0.007)	0.079^c^ (±0.005)	4.26^d^ (±0.34)	4.04^d^ (±0.18)

T1-Irrigated control, T2-Untreated plants grown under rainfed conditions, T3-Coinouclation of *P*. *chinense* and *B*. *cereus*, T4-Consortium of PGPR and SA, T5-SA only. Different letter shows that the difference is statistically significant (*P* < 0.05) within treatments and ± represents standard error values.

## Discussion

Moisture stress is one of the major impediments to agricultural productivity. Approximately, 40% of agricultural lands are rainfed, which can lead to moisture stress and its attendant decreases in plant nutrient uptake and transport. Plant growth promoting rhizobacteria (PGPR) in combination with salicylic acid (SA), could play a critical role in the mitigation of moisture stress in plants. PGPR consortium superseded the effects of the PGPR consortium in addition with SA on sugar, protein and proline production in plant leaves. However, SA spray applied to the PGPR inoculated plants improved the efficiency of the PGPR for chlorophyll fluorescence, PI, and phenolic content. As a result of the higher phenolic content, plant productivity was improved along with the induction of resistance to pathogen attacks.

The exopolysaccharides produced by these PGPR were shown to have profound effects on plant growth and drought tolerance. Exopolysaccharides are responsible for maintaining higher moisture content and growth of plants under severe rainfed condition. They form a rhizosheath around the roots and thus protect the plant roots from desiccation for a longer period of time. Important role exhibited by exopolysaccharides includes, protection from desiccation, microbial aggregation, plant-microbe interaction, surface attachment and bioremediation. Plants inoculated with EPS-producing bacteria showed higher accumulation of proline, sugars, and free amino acids under water deficit stress [51]. Plants treated with EPS-producing bacteria *Azospirillum* showed resistance to water stress through improvement in the soil structure and soil aggregation [52]. Under drought stress, inoculation of sunflower with EPS-producing bacterial strain YAAF34 showed increase in root tissue [53]. Seed bacterization of maize with EPS-producing bacterial strains in combination with their respective EPS improved soil moisture contents, plant biomass, root and shoot length, leaf area and leaf protein and sugar contents under drought stress condition [54]. Khan *et al*. [31] reported similar results in wheat plants grown under sandy soil condition.

Kumar *et al*. [55] reported a decrease in chlorophyll content following drought stress. In this study the effect of PGPR alone was on par with that of SA applied alone, whereas, the addition of SA to the PGPR helped equalize or even increase the chlorophyll content versus the untreated unstressed plants grown under irrigated conditions. This indicated that the SA and PGPR interacted synergistically to effectively ameliorate the antagonistic effects of moisture stress on chlorophyll production and fluorescence. The combined effect of PGPR plus SA improved the water budget of the plant as demonstrated by the augmented fresh and dry weight of plants and the RWC of the leaves.

PGPR enhancement of chlorophyll content in many plants grown under abiotic stress conditions has been previously described [56, 57]. SA delays and prevents the degradation of chlorophyll that results from abiotic stresses [58–61]. The present study demonstrated that SA combined with PGPR helped both genotypes to maintain an efficient photosystem with an improved water budget, resulting in improved growth and productivity under rainfed conditions.

New proteins appeared to be synthesized in stressed plants, and the tolerant variety had greater potential and produced more protein to better cope under stress conditions. Dashti *et al*. [62] indicated that co-inoculation of soybeans with *B*. *japonicum* and *Serratia species* increased grain yield, protein yield, and total plant protein content. Afzal and Bano [63] reported a PGPR-induced increase in the leaf protein content of wheat. Similar results were reported by Islam *et al*. [64] and Pérez-Montaño *et al*. [65] in cereal and leguminous plants. The additive effect of SA on leaf protein content had been reported previously by Neelam *et al*. [66] and Çanakci and Dursun [67].

PGPR-induced accumulation of soluble sugars leads to drought tolerance in plants as the sugars act as an osmoprotectant under water stress conditions. Soluble sugars act directly as negative signals, or modulators, of plant sensitivity and, thus, they can also play an important role in cell responses to stress-induced remote signals [68–70]. Plant phenols help plants survive under severe stress conditions. Coinoculation of 2-PGPR and SA significantly augmented the plants’ phenolic content and, hence, increased their tolerance to moisture stress. These results were in accord with those described by Bahadur *et al*. [71] and Chakraborty *et al*. [72], which demonstrate a rise in phenolic content in the leaves of pea plants inoculated with PGPR strains. SA treatment significantly enhanced plant phenolic content [73, 74].

A positive correlation exists between proline accumulation and plants exposed to different stresses. Proline imparts stress tolerance to plants by maintaining cell turgor or osmotic balance, and stabilizing membranes thereby preventing electrolyte leakage, and bringing concentrations of reactive oxygen species (ROS) within normal ranges. In turn, this prevents oxidative damage in plants. Accumulation of proline normally occurs in the cytoplasm, where it functions as a molecular chaperone, stabilizing the structure of proteins. Further, its accumulation buffers cytosolic pH and maintains cell redox status [75–77]. Proline production was significantly enhanced in plants grown under rainfed conditions. However, the application of PGPR helped the plants cope with osmotic stress and maintained the bioenergetics of the plant cells under stress; thus, it did not cause any substantial increase in proline production, as no extra proline was required. The decline in proline production was further augmented by the combined treatment of SA and PGPR, keeping it on par with the irrigated controls, thereby, demonstrating that the PGPR combination induced the ability for the plants to overcome osmotic adjustment. Jha *et al*. [75] demonstrated that proline accreted in higher concentrations with an increase in salinity, but declined in plants inoculated with PGPR alone.

The ameliorative effects of PGPR is worth mentioning for the lipid peroxidation as measured by the malondialdehyde (MDA) content of the leaves. It is inferred from the result that similar characteristic exist for SA. Lipid peroxidation act as biomarker for tissues and membrane damage under stress condition [76, 77]. Increase in lipid peroxidation is considered as an indication for increase in oxidative damage. Singh and Jha *et al*. [78], documented a rise in lipid peroxidation with the increase in salt concentrations in wheat, however, PGPR treatment significantly reduced the lipid peroxidation in salt treated plants. This reduction in lipid peroxidation with PGPR-inoculation was due to the fact that PGPR inoculation lower cell injuries caused by abiotic stresses and increase tolerance to environmental stresses. Coinoculation of *Pseudomonas pseudoalcaligenes* and *Bacillus pumilus* had significant adverse effects on lipid peroxidation in paddy grown under salt stress condition [79]. PGPR mediated decreases in lipid peroxidation had been reported previously in other plants [72, 73], but their effect in combination with SA has not been studied. PGPR plus SA treatment ameliorated drought induced increase in lipid peroxidation in both the genotypes.

Antioxidant enzymes play a critical role in detoxifying the destructive effects of reactive oxygen species, created in plants under moisture stress in rainfed areas. The observed decrease in antioxidant enzyme activities with PGPR-inoculation may be credited to the fact that PGPR ameliorate the adverse effects of moisture stress hence, reducing the production of reactive oxygen species. These results were in covenant with those stated by Jha and Subramanian [79], who stated that PGPR-inoculation reduced the lipid peroxidation and SOD activity in sensitive cultivars of *Oryza sativa* and endorsed resistance to salt stress. Exogenous application of SA was reported [80–83] to regulate the activities of different enzymes such as SOD, POD, CAT, APOX and in turn enhance tolerance to various stresses.

Relative Water Content reflects a measure of plant water status, which in turn is used as an index for dehydration tolerance [84–87]. Significant decrease in Relative Water Content in untreated uninoculated plants grown in rainfed area was observed but the percent decrease was much higher (43%) in the sensitive genotype. The coinoculation of 2-PGPR (*P*. *chinense* and *B*. *cereus*) alone or in combination with SA had marked improvement on plant biomass. The treatments were more responsive in sensitive genotype than tolerant one except for shoot dry weight. Ahemad and Kibret [88] reported enhanced shoot fresh and dry weights in *Brassica* plants inoculated with PGPR. Similar results were reported by Islam *et al*. [89] in cucumber and by Huang *et al*. [90] in corn, pepper and tomato. The effect of SA on shoot fresh and dry weights was more pronounced. SA induced increase in shoot fresh and dry weights had been well documented in many plants including, ocimum, lemongrass, sunflower, strawberry, wheat and maize [91].

## Conclusion

It is concluded that the combined effect of SA and PGPR significantly overcome drought induced increase in antioxidant enzymes, proline production and lipid per oxidation of leaves. These results indicate that the differential accumulation of these metabolites lead to drought tolerance mechanism even in sensitive genotype as sensitive genotype also showed altered levels of different metabolites. The PGPR and/or SA showed increase in all growth parameters but the magnitude of increase was higher in combined treatment of PGPR plus SA. The greater decrease in soil moisture content was in the sensitive genotype for the measurement made at harvest. The effect of PGPR consortium was par with that of PGPR consortium plus SA treatment but it is quite evident that SA act synergistically with that of PGPR. The observed increase in RWC of plant leaves among PGPR and SA treatments may be attributed to the PGPR plus SA induced increase in soil moisture. The increased availability of the soil moisture and enhanced RWC of leaves augmented accumulation of macro/micronutrients e.g. Ca, Mg, Na and K. The data demonstrate that SA applied singly has more pronounced stimulatory effects on phenolic contents of leaves, shoot dry weight and Zn accumulation over control. Integrative use of active PGPR strains (biotic elicitors) and SA seems to be a promising approach and eco-friendly strategy for increasing drought tolerance in plants.
